# Using machine learning tools to predict outcomes for emergency department intensive care unit patients

**DOI:** 10.1038/s41598-020-77548-3

**Published:** 2020-12-01

**Authors:** Qiangrong Zhai, Zi Lin, Hongxia Ge, Yang Liang, Nan Li, Qingbian Ma, Chuyang Ye

**Affiliations:** 1 Department of Emergency, Peking University Third Hospital, 49 North Garden Rd, Haidian District, Beijing, China; 2 Institute of Signal and Image Processes, Beijing Institute of Technology, 5 South Zhongguancun Street, Haidian District, Beijing, China; 3 Research Center of Clinical Epidemiology, Peking University Third Hospital, Beijing, China

**Keywords:** Outcomes research, Translational research

## Abstract

The number of critically ill patients has increased globally along with the rise in emergency visits. Mortality prediction for critical patients is vital for emergency care, which affects the distribution of emergency resources. Traditional scoring systems are designed for all emergency patients using a classic mathematical method, but risk factors in critically ill patients have complex interactions, so traditional scoring cannot as readily apply to them. As an accurate model for predicting the mortality of emergency department critically ill patients is lacking, this study’s objective was to develop a scoring system using machine learning optimized for the unique case of critical patients in emergency departments. We conducted a retrospective cohort study in a tertiary medical center in Beijing, China. Patients over 16 years old were included if they were alive when they entered the emergency department intensive care unit system from February 2015 and December 2015. Mortality up to 7 days after admission into the emergency department was considered as the primary outcome, and 1624 cases were included to derive the models. Prospective factors included previous diseases, physiologic parameters, and laboratory results. Several machine learning tools were built for 7-day mortality using these factors, for which their predictive accuracy (sensitivity and specificity) was evaluated by area under the curve (AUC). The AUCs were 0.794, 0.840, 0.849 and 0.822 respectively, for the SVM, GBDT, XGBoost and logistic regression model. In comparison with the SAPS 3 model (AUC = 0.826), the discriminatory capability of the newer machine learning methods, XGBoost in particular, is demonstrated to be more reliable for predicting outcomes for emergency department intensive care unit patients.

## Introduction

As the number of critically ill patients has increased in emergency departments (ED) globally^1^, the demand for critical care has also increased substantially in the last decade, and exceeds capacity in many systems^2^. Capacity is challenged because critical care is an expensive and limited resource, and critically ill patients should be admitted to the intensive care unit (ICU) without delay^3^. However, as the number of critically ill ED patients continues to increase, their stay in the ED has become even longer^4^. Because of the scarcity of ICU beds, many EDs are in the process of changing toward providing units capable of delivering critical care, known as ED intensive care units (ED-ICU). After providing resuscitation and stabilization, critical care of the patients is continued in the ED-ICU^5^. Staffing and resources are also, however, limited in emergency departments, and the ED-ICU patient population features diseases that differ in proportion from the general intensive care population. There are certain clinical scenarios in which the ED intensivist’s unique skill set can positively affect patient care, but studies have shown that ED-ICU patients have a higher unadjusted mortality than non-ED ICU patients. Traditional ICU scoring systems do not take emergency patients’ characteristics into consideration. Accurately assessing the severity of critically ill patients and predicting adverse outcomes are therefore important for initial triage and treatment in the ED^6^. Identifying non-survivals precisely is important not only because it provides information needed for avoiding unnecessary invasive care, but it also gives physicians credible evidence that could aid in the timely start of palliative care. Moreover, the early prediction of these cases helps the patients’ relatives prepare mentally and financially.

Several studies report scoring systems that can be applied in the ED, such as the DAVROS project^7^. However, the project was designed for all emergency department patients, not just the critically ill and injured. Both the derivation and validation cohorts in the project had an average mortality ranging from 4.2 to 6.9%, which is less than the ED-ICU population. On the other hand, the Simplified Acute Physiology Score (SAPS) model was developed specifically for ICU scoring, and was revised to SAPS 3 in 2005 in order to develop a new, improved model for risk adjustment^8^. Although robust illness severity scoring systems such as the SAPS have been developed and validated in the ICU setting, their predictive value is substantially degraded when applied to the rapidly changing physiology of an ED patient during the first several hours of resuscitation and critical care management. Given these limitations, it is clear that an outcome prediction tool optimized for the unique ED-ICU patient population is an essential foundation for future clinical research and practice.

In recent years, clinical use of machine learning has been evolving rapidly as opposed to typical clinical algorithms that often consist of handcrafted rules with numerous exceptions. The machine learning technique has been identified as a robust and reliable tool in predicting outcomes. Yet established methods become numerically unstable with large sets of predictors and their interactions^9^. Our objectives are to construct four machine learning models to predict the mortality of ED-ICU patients and compare their prediction performance.

## Results

**Figure 1:**
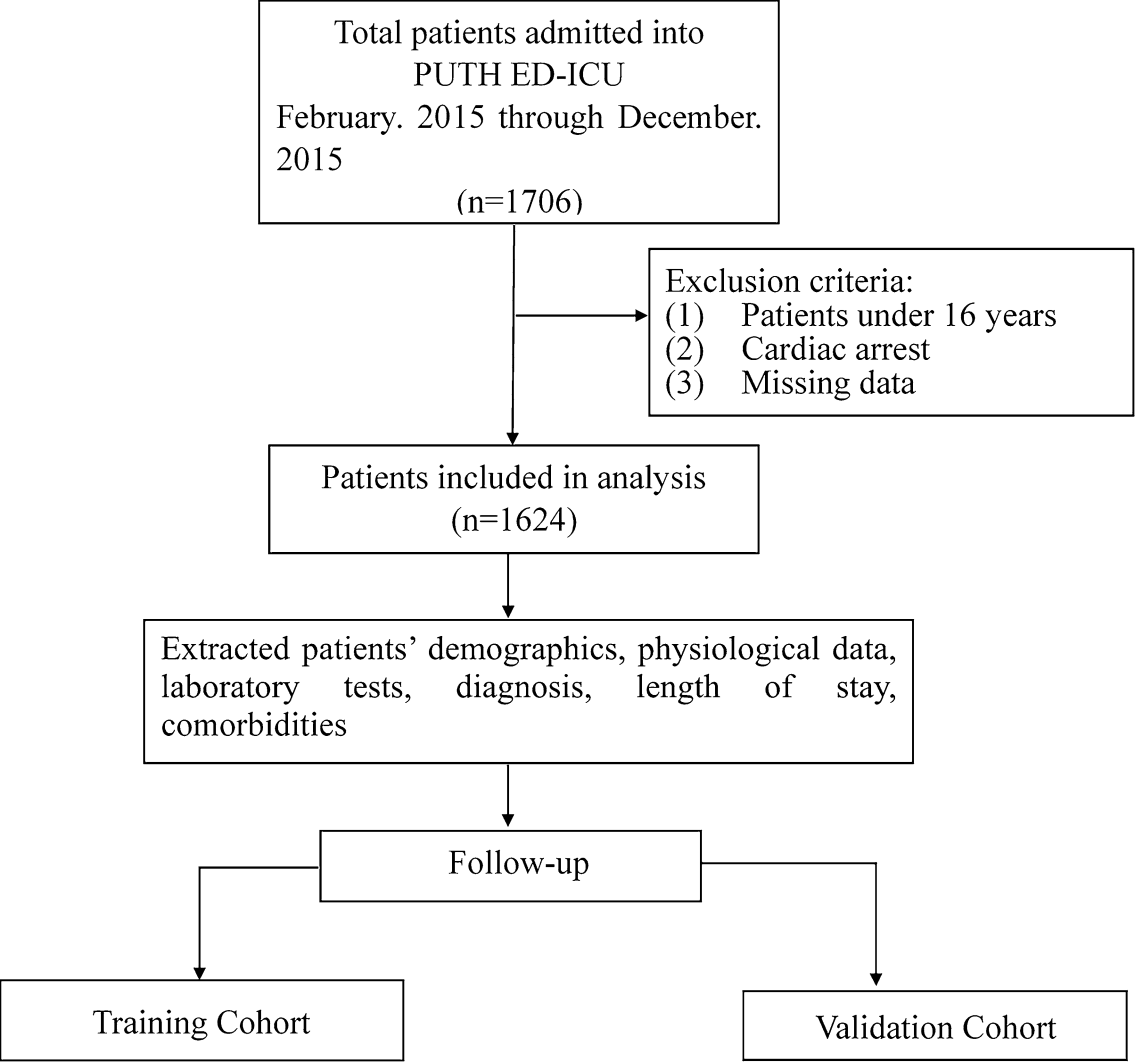
Schematic diagram of the present study.

As shown in Fig. 1, 1624 cases were included in the final analysis. Of the included patients, 60% were male, and the mean age was 64.7 ± 18.1 years. Table 1 details the demographics, physiological characteristics, and laboratory results of the studied population, divided according to survival status. Significant differences were observed in age and laboratory results. Univariate analysis showed non-survivors were more likely to be older, and to have lower Glasgow coma scores, higher respiratory rates and systolic blood pressure (all *p* < 0.001). The classes imbalance was analyzed and it shows no effect on the result. (Supplementary Material-Fig. 1).

**Table 1:** Baseline characteristics of study population.

Characteristic	Total (n = 1624)	Survivors (n = 1413)	Non-survivors (n = 211)	*p*
**Demographics**				
Male, n (%)	969 (60.0)	844 (59.7)	125 (59.2)	0.91
Age, years	64.7 ± 18.1	63.7 ± 18.3	71.2 ± 14.9	< 0.001*
**Physiologic characteristics**				
Glasgow coma scale	15 (15–15)	15 (15–15)	15 (7–15)	< 0.001*
Respiratory rate (breaths/min)	22.0 ± 7.0	21.6 ± 6.6	24.3 ± 6.6	< 0.001*
Heart rate (beats/min)	96.8 ± 30.5	96.4 ± 30.6	99.7 ± 32.5	0.147
Systolic blood pressure (mm Hg)	133.4 ± 31.1	134.4 ± 29.6	127.1 ± 39.8	0.002*
**Laboratory results**				
White cell count (10^9^/L)	9.4 (7.0–13.0)	9.1 (6.9–12.3)	12.6 (8.8–17.0)	0.007*
Platelet count (10^9^/L)	213.7 ± 93.5	217.2 ± 91.5	189.9 ± 102.7	< 0.001*
Hemoglobin (g/L)	124.7 ± 31.3	126.5 ± 30.6	112.1 ± 33.3	< 0.001*
Serum potassium	4.2 ± 0.8	4.1 ± 1.8	4.4 ± 1.1	< 0.001*

*The difference between the survivor and non-survivor groups was statistically significant.

**Figure 2:**
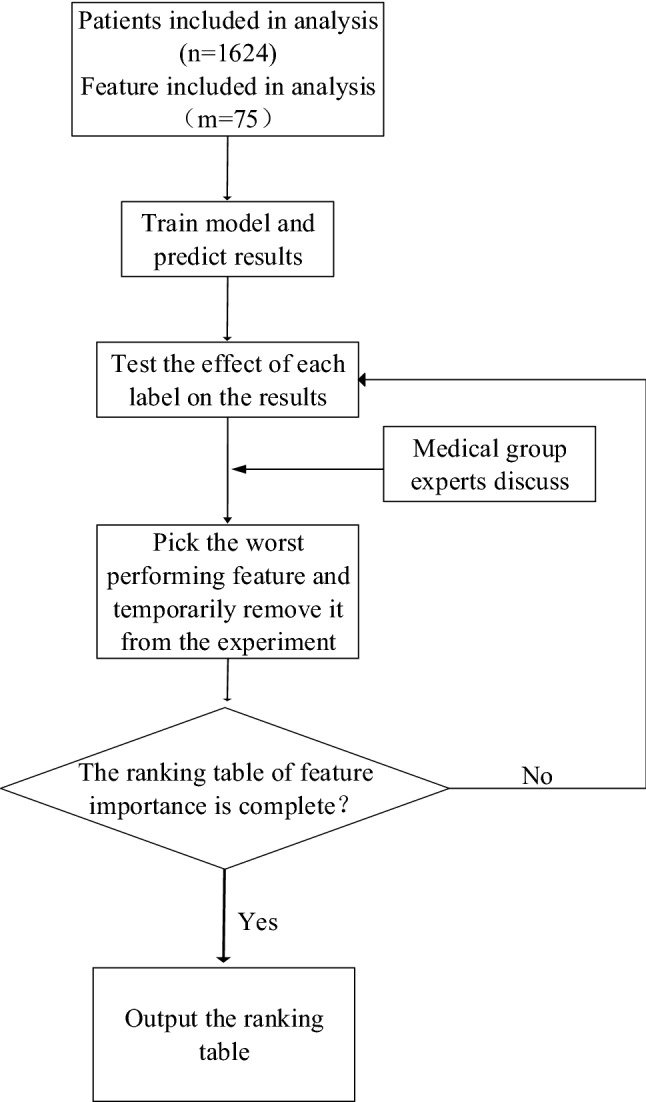
Feature selection steps adopted by machine learning.

In the feature selection phase of the experiment, the dataset was ultimately divided into three groups. First it was divided into a test set (20%) and feature selection set (80%); Then the feature selection set was randomly divided into a training set (50% of the full dataset) and a validation set (30% of the full dataset), for the XGBoost training model and testing model, respectively. AUC values were used to measure the influence of each feature in each feature group in the model, and features with the least influence in each group were removed from the feature group. This step was repeated until all features were ranked by their importance. Figure 2 shows the process for determining the importance score of a feature. In a single experiment, each of the 75 features were ranked on a scale of 0 to 75 in order of importance, with 75 being the least important and 0 being the least. After ten experiments and feature scoring, we obtained the final ranking by synthesizing the scoring of all experiments. The importance ranking of all the features is shown in Table 2.

**Table 2:** Importance ranking of all features.

	Feature name	Total score
1	GCS score first	683
2	Hemoglobin	670
3	Glucose	639
4	FiO2 first	622
5	Planed admission	600
6	BUN	591
7	Septic shock	584
8	Shock first	577
9	White Blood Cell	569
10	Systolic blood pressure first	553
11	Cancer therapy	551
12	Respiratory rate first	543
13	Disch dx cerebrovas	534
14	Metastatic cancer	505
15	Sodium	505
16	SpO_2_ first	503
17	pO_2_ first	501
18	Platelet	498
19	Disch dx neoplasms	471
20	Tbil	471
21	Agitation	470
22	pH first	467
23	Coma	463
24	Disch dx digestive disease	450
25	Palpitation	445
26	Altered mental status	434
27	Potassium	434
28	Acute abdomen	425
29	Disch dx circulatory disease	422
30	Creatinine	421
31	Heart rate first	420
32	Active malignancy	403
33	Dyspnea	393
34	Severe acute pancreatitis	392
35	O_2_ flow rate first	391
36	Fever	389
37	Age	385
38	Hypovolemic hemorrhagic shock	367
39	Focal neurologic deficit	356
40	Intracranial effect	356
41	Obtunded	350
42	Disch dx flu pneumonia	347
43	Disch dx gu disease	347
44	Confusion	342
45	Arrhythmia	337
46	Cirrhosis	328
47	Stupor	325
48	Vigilance disturbance	325
49	Anaphylactic shock	317
50	Disch dx resp	312
51	Disch dx other disease	307
52	Hypovolemic non-hemorrhagic shock	305
53	Disch dx chronic lower resp	304
54	Seizures	303
55	Live failure	299
56	O_2_ device first	299
57	Vomiting	293
58	Mix shock	289
59	Use vasoactive drugs	278
60	Hematemesis	272
61	Infection	268
62	Disch dx aids	258
63	Steroid therapy	248
64	Chronic heart failure iv	231
65	Chest pain	208
66	Fatigue	192
67	Disch dx abnormal nos	187
68	Hematologic cancer	174
69	Disch dx injury	169
70	Trauma	147
71	Syncope	143
72	Bloody stools	138
73	Headache	124
74	Abdominal pain	120
75	Chest tightness	86

**Figure 3:**
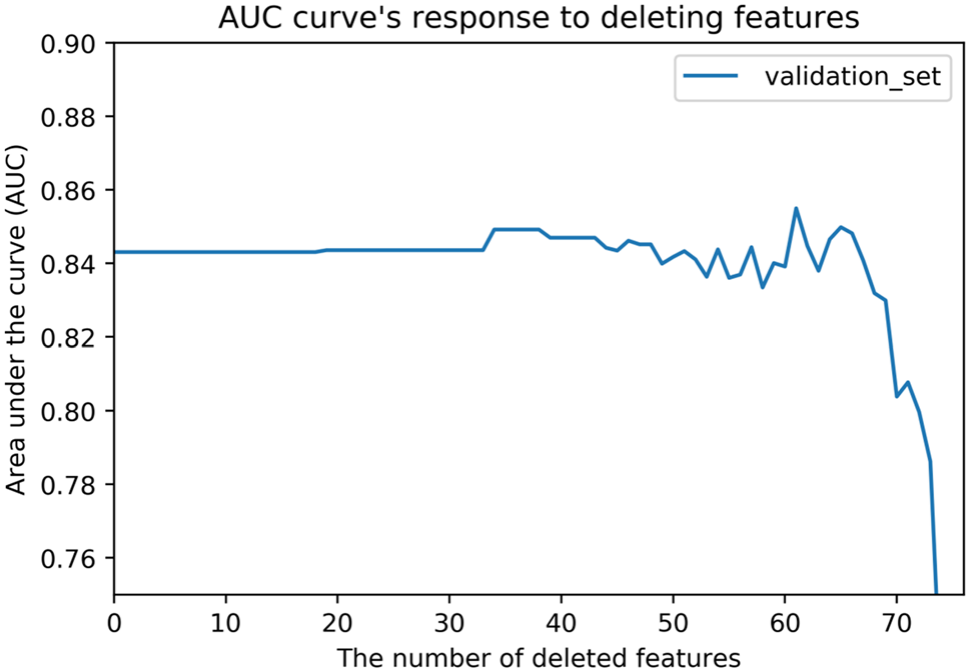
AUC curve for feature selection. The curve of the test set shows that the obtained feature importance table can play a role in optimizing the results for a lightweight model.

**Figure 4:**
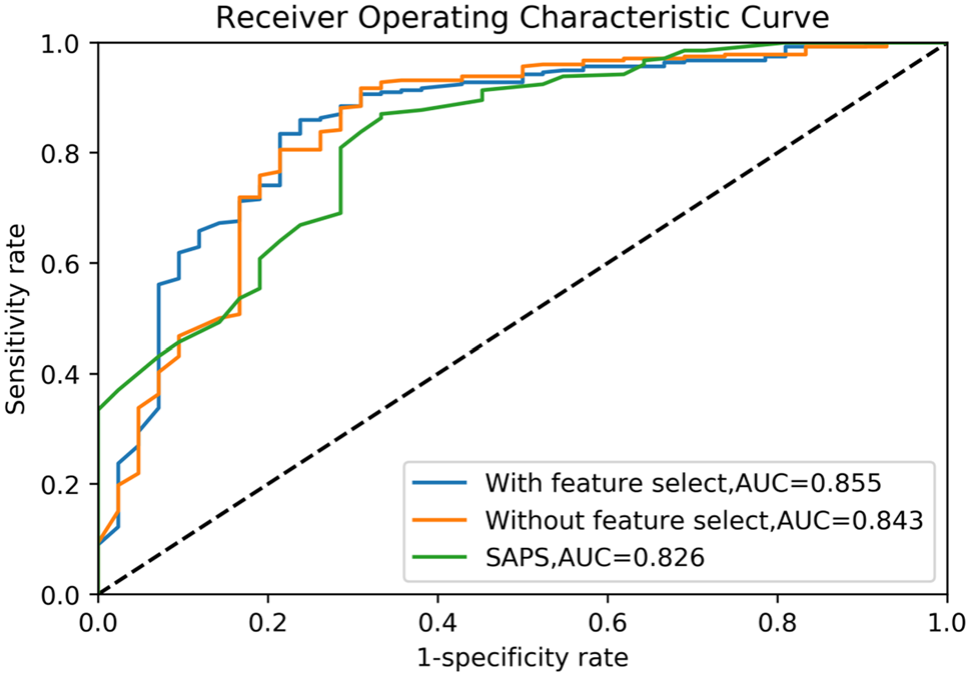
ROC curve for the three methods. By observing the ROC curves of the three methods, it is obvious that the machine learning method has better performance than the traditional SAPS-3 scoring method, and the machine learning model after feature selection is superior.

**Figure 5:**
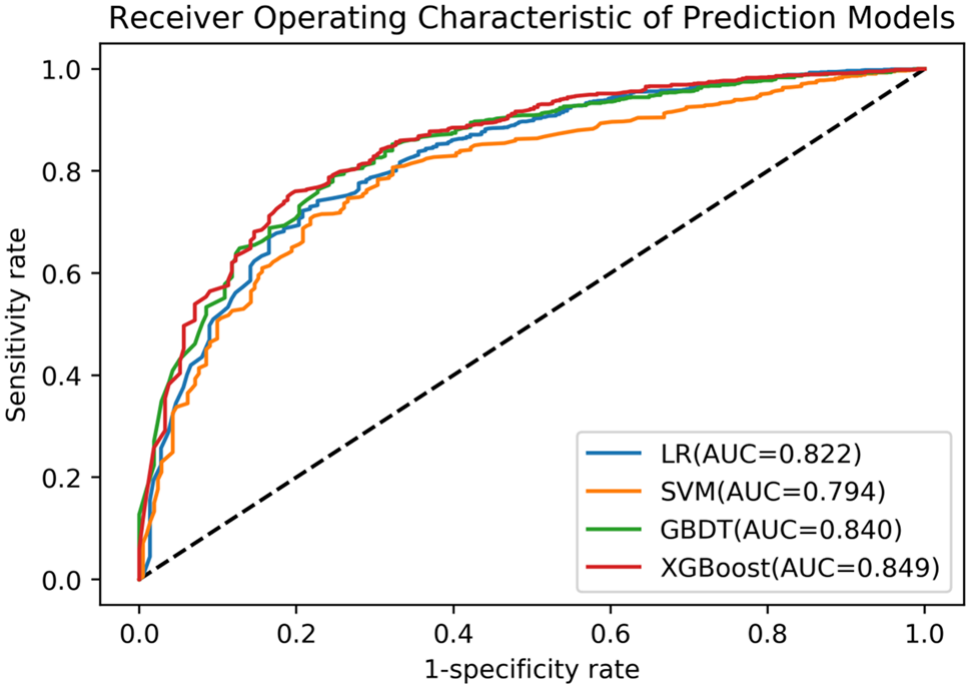
ROC curves for the four predictive models. The ROC curve of XGBoost is superior to other methods and has the best AUC index performance.

In order to verify the accuracy of the feature importance table, we used the feature selection set to train the model and tested it on the test set. Then we sorted the features according to their importance in Table 2, successively deleted the features with lower scores, and recorded the changes in AUC on the test set. The AUC variation curve with feature selection is shown in Fig. 3. Figure 4 shows the ROC curves of the model with and without feature selection. Through the analysis shown in Figs. 3 and 4, we find that our XGBoost machine learning model is still stable on the test set after the deletion of 50 features, and in some cases the model becomes more accurate due to the deletion of redundant information. Its predictive value is better than that of SAPS3. As the features continue to be deleted, the machine learning model based on just 13–15 features still has relatively reliable performance. By combining medical evaluation with machine learning, we attained a lightweight, high-quality predictive model. The AUC values are shown in Fig. 5, and the other indicators in Table 3. According to the table, we concluded that XGBoost had the best performance for our dataset in the three aggregate metrics. The XGBoost algorithm was therefore used to further analyze the selected features.

**Table 3:** Predictive accuracy for the four predictive models.

Method	Sensitivity	Specificity	Overall accuracy	AUC	Youden Index (Se + Sp-1)
LR	0.742	0.772	0.832	0.822 (0.72–0.85)	0.514
SVM	0.712	0.777	0.802	0.794 (0.72–0.86)	0.489
GBDT	0.790	0.754	0.834	0.840 (0.76–0.88)	0.543
XGBoost	0.756	0.806	0.837	0.849 (0.81–0.89)	0.562

## Discussion

Our primary goal in this study has been to accurately identify high-mortality patients as to best utilize emergency department resources. ED-ICU patients, as a unique subgroup of ICU population, are characterized by their uncertainty and rapid deterioration. The situation calls for an automated system to generate risk prediction in real time. Even though there are a number of risk adjustment algorithms available, the utilization rate remains low for ICU patients^10^. Heterogeneous variables combined with complicated mathematical calculation make implementation of traditional algorithms impractical. Previous studies have focused on using machine learning methods to predict outcome^11–13^, and have shown that a machine learning approach could potentially outperform existing traditional analytic techniques for predicting in-hospital mortality of ED patients with sepsis^14^, coronary artery disease^15^ and critical care patients^16^.

It has been suggested that many aspects of patient care and assessment are ‘‘pattern recognition’’ tasks that could benefit from machine learning-based prediction models and application^17^. Machine learning models have several advantages over other methods. For example, they are adept at handling high-order interactions and non-linear relationships between the predictors and the outcome^18^. Machine learning techniques may be optimized and combined into a multimethod solution given the right parameters and performance criteria.

In this study, we tested several machine learning algorithms for predicting outcomes in critically ill emergency patients. Our models were built on original data that required few customization, so any medical institution that has access to electronic clinical and administrative systems can employ the method readily. Traditional predictive systems such as the APACHE risk adjustment algorithm focus on the most serious physiological parameters; in comparison, our models are fully automated, so they can consider all information to generate results. We found machine learning to be a favorable prediction tool, especially the XGBoost method; as shown in other research^19,20^, predictive tools using XGBoost showed excellent performance in comparison to other algorithms. XGBoost combines weak learners (decision trees) to achieve stronger overall class discrimination^21^. Compared with gradient boosting, XGBoost can be more efficiently parallelized, and incorporates regularization and tree pruning to reduce overfitting^22^.

The feature selection process is important in developing the model because building a machine learning tool based on all the clinical variables is not feasible. Critical disease prediction involves many more variables than do predictive systems for other diseases. Current approaches incorporate only the essential variables based on physicians’ experience, while the machine learning methods used in this study were not limited to a small set of risk factors. However, expanding the spectrum of variables does not mean machine learning methods require all details to yield results. The XGBoost included 89 features to reach a conclusion, but it is also acceptable to input a dozen essential variables to obtain a reasonable result. Given the breadth of options, the first feature screening reduced the number to 75 features. The ranking order showed many similarities with other well-recognized scoring systems, such as the SAPS system. The presence of shock and altered mental status, for example, was heavily weighted in both systems, showing the machine learning method coinciding with physicians’ experience. With the help of a developed electronic health record database, it is feasible for our tool to generate real-time predictive value, which may help physicians to evaluate the effects of treatment. In comparison, traditional scoring systems often require data to be complete and restrictive in order to achieve fixed outcomes.

To our knowledge, this is the first published study that has applied machine learning methods to predict critically ill emergency patients. Machine learning ensured the appropriate evaluation of variables as well as accurate results. Nevertheless, several limitations exist in this study. This was a single center, retrospective study. External validation is still needed, and adequate validation in other centers is planned for the future. Extended follow-up is also needed for improving predictive applicability. In addition, machine learning techniques have been criticized as black boxes, and thus may be viewed with suspicion by clinicians. Chen and Asch^23^ observed that while machine learning identifies factors predictive of mortality, such as palliative care, ending such care to reduce mortality would be irrational. This study has addressed this concern by adding the physician feature screening process to avoid building a model with such irrational variables. With the help of experienced clinicians, we have established a sound foundation for deriving a useful model, and hope that it will be applied in clinical practice in the future.

## Methods

### Setting, participants, and data collection

A retrospective cohort study was performed in the Peking University Third Hospital. The facility’s emergency system consists of an 18-bed resuscitation unit and a 15-bed ED intensive care unit. Patients were eligible for inclusion in the study if they were alive on emergency medical services (EMS) arrival and were admitted to the resuscitation unit or the ED-ICU between February 2015 and December 2015. The exclusion criteria are presented in the Fig. 1. The institutional Ethics Committee (Peking University Third Hospital Medical Science Research Ethics Committee) approved this study, and issued a waiver of informed consent since all examinations were part of standard patient care, and, since the study was retrospective, there was no interference with patient treatment.

An expert panel consisting of emergency physicians and epidemiologists was established to design the study. Before initiation of the study, the panel developed a standard searching strategy to be applied by researchers to avoid inconsistency. Data from the electronic medical records was extracted by several researchers who are physicians well-trained in resuscitation. Extracted data included patients’ demographics, comorbidities, physiological data, laboratory tests, diagnosis, and length of stay. The variables were restricted to those collected within 6 h of medical contact. All data were entered into a secure database managed by the research team who, from the medical records, identified death within seven days as the primary outcome.

### Imputation of missing data

In the data set used for this study, some data was incomplete (even when we have already excluded patients with too much missing data). Since the missing proportion of the collected features was less than 5%, we chose to replace the missing values with the average of that variable based on the patient data that was complete.

### Feature screening process

There are a lot of clinical variables are available in the Peking University Third Hospital health records database, and many of the variables are repetitive and not well organized. To build the optimum machine learning model, candidate variables were examined and discussed extensively in team meetings before they were entered into the four models. Existing risk adjustment algorithms and published ICU admission criteria (mainly DAVROS and SAPS 3) provided the basis for screening the variables. Additionally, the Delphi method and literature review were used in order to determine the effect every variable, or feature would have on the models. Each variable was evaluated based on its significance, representativeness, and accessibility. At the end of this initial screening process, 75 features were selected.

### Model development

This study used the Python 2.7 (Anaconda) platform to train the model with the scikit-learn 0.19.1 framework. Python 2.7 makes it easy to create experiments and debug different machine learning frameworks. Four model types were compared:

*Logistic regression*^7^ logistic regression (LR) is a generalized logistic regression analysis model that is often used in data mining, automatic disease diagnosis, economic forecasting, and other applications. Using binary logistic regression, univariate analyses were performed on all 75 variables of interest to explore their association with death for all patients. Variables identified by univariate analysis to have a p value of less than 0.05 were considered to have clinical significance. Multivariate analyses were then performed on that group of variables to further explore their association with death. For example, the model can be used to explore risk factors related to a particular disease, and predict the probability of the disease occurrence based on presence of the risk factors. In this study, two groups of subjects were selected, one of which was constituted of death cases within seven days after cardiac arrest treatment, and the other constituted of survival cases. The two groups represented the dependent variable, that is, whether the patient was alive after 7 days. Since the two groups had different physiological characteristics and habits, the 75 independent variables included various types of patient features, either continuous or categorical. Through logistic regression analysis, the weight of the independent variables was obtained in order to determine which of the features were risk factors. At the same time, the weight value of the risk factors was able to predict the possibility of a patient's death.

*Support vector machine*^7^ Support Vector Machine (SVM) is a data mining method based on statistical learning theory. Its principle is to find an optimal classification hyperplane that meets the classification requirements^21^ so that the hyperplane can maximize the margins on both sides while ensuring classification accuracy. In theory, support vector machines can achieve optimal classification of linearly separable data.

*Gradient boosting decision tree* Gradient Boosting Decision Tree (GBDT) is one of the best machine learning algorithms for fitting real distributions, as it combines boosting and decision tree algorithms. The decision tree is a basic classification and regression tree algorithm (CART), which has the advantages of fast classification and visualization of the model. Boosting learns multiple classifiers by increasing and reducing the weight of the training samples, and linearly combining these classifiers to improve performance. The main idea is that each time the model is built, the gradient direction of the model loss function is established, so that the loss function decreases along the gradient direction. In summary, the GBDT algorithm uses a gradient descent algorithm to train multiple learners for complex tasks, and then combines the results of multiple learners to obtain a better classification result than it could using a single learner.

*XGBoost*^21^ XGBoost, which stands for extreme gradient boosting, is an improved form of GBDT. The traditional GBDT algorithm uses CART as the base classifier, and XGBoost also supports linear classifiers. However, the traditional GBDT algorithm only uses first-order derivative information in optimization, whereas XGBoost performs second-order Taylor expansion for minimizing the cost function, and uses first-order and second-order derivatives. To control the complexity of the model, XGBoost adds to the cost function a regularization term that contains both the number of leaf nodes of the tree and the sum of the squares of the L2 norms of each leaf node’s score output. The regularization term thus reduces the variance of the model, making the learned model simpler and preventing overfitting. XGBoost also supports column sampling in random forests, which not only reduces overfitting but also reduces computation overhead. These characteristics make XGBoost an improvement over the traditional GBDT algorithm and have made it one of the most popular machine learning algorithms.

The LR, SVM and GBDT algorithms were implemented by directly using the package in scikit-learn 0.19.1^7^. To implement XGBoost, the XGBoost 0.82 framework was integrated with scikit-learn 0.19.1. For parameter settings, we chose L1 regularization for the LR, and linear kernel for the SVM. The parameters for the GBDT and XGBoost algorithms were set to default.

### Model comparisons

To compare the performance of the LR, SVM, GBDT, and XGBoost algorithms, we used ten-fold cross-validation; that is, we randomly divided the patient data (the 75 selected features) into ten folds and conducted ten experiments. In the i-th (i = 1, …, 10) experiment, we used the i-th fold of data as the test set, and the remaining data as the training set. The mean value of the results of all experiments was computed to measure the accuracy of the algorithm. The average accuracy (ACC), sensitivity (Se), specificity (Sp), Youden index, and area under the curve (AUC) of each of the models were established by the 10-fold cross-validation.

### Statement

The authors must identify the committee that approved the research. We confirm that all research was performed in accordance with relevant regulations. The institutional Ethics Committee approved the study and issued a waiver of consent.

## Supplementary information

Supplementary Information.
