# Supplementary material for: Using machine learning tools to predict outcomes for emergency department intensive care unit patients
Source: Sci Rep. 2020 Dec 1;10:20919. doi: 10.1038/s41598-020-77548-3 (PMC7708467; doi:10.1038/s41598-020-77548-3)
Supplement: Supplementary file 1 — Supplementary Information. [file 41598_2020_77548_MOESM1_ESM.docx]

**Using Machine Learning Tools to Predict Outcomes for Emergency Department Intensive Care Unit Patients**

**Supplementary Material**

Corresponding Author: **Qingbian Ma, MD^a^; Chuyang Ye, Ph.D^b^**

Co-Authors: Qiangrong Zhai, MD^a#^; Zi Lin^b#^, Hongxia Ge, MD^a^; Yang Liang, MD^a^; Nan Li, Ph.D^c^, Qingbian Ma, MD^a^, Chuyang Ye,Ph.D^b^；

^a^Department of Emergency of Peking University Third Hospital, 49 North Garden Rd, Haidian District, Beijing, China

^b^Institute of Signal and Image Processes, Beijing Institute of Technology, 5 South Zhongguancun Street, Haidian District, Beijing,China

^c^ Research Center of Clinical Epidemiology, Peking University Third Hospital, Beijing, China.

^#^ Contribute equally to the manuscript

Correspondence to: Qingbian Ma at [maqingbian@126.com](mailto:maqingbian@126.com)

Chuyang Ye at chuyang.ye@bit.edu.cn


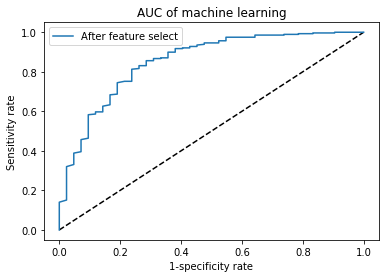


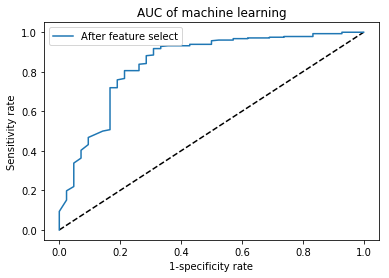


Figure 1. The resample of data. A. AUC=0.8523 after resample. B.AUC=0.8433 without resample
